# Serum Interleukin-34 Levels Are Elevated in Patients with Systemic Lupus Erythematosus

**DOI:** 10.3390/molecules22010035

**Published:** 2016-12-28

**Authors:** Hongxu Wang, Ju Cao, Xiaofei Lai

**Affiliations:** Department of Laboratory Medicine, The First Affiliated Hospital of Chongqing Medical University, Chongqing 400016, China; wanghx502@163.com (H.W.); caoju723@163.com (J.C.)

**Keywords:** systemic lupus erythematosus, SLE, interleukin-34, IL-34, activity index, immune biomarker

## Abstract

Interleukin-34 (IL-34) was initially identified as an alternative ligand for the colony-stimulating factor-1 receptor (CSF-1R) to mediate the biology of mononuclear phagocytic cells. Recently, IL-34 was found to be associated with chronic inflammation, such as in rheumatoid arthritis (RA). Both RA and systemic lupus erythematosus (SLE) are multifactorial autoimmune diseases and are characterized by excessive immune and inflammatory responses. Thus, we investigated whether IL-34 is involved in the pathogenesis of SLE. In all, 78 SLE patients and 53 healthy controls were enrolled in the research. Enzyme-linked immunosorbent assay (ELISA) was employed to measure the concentrations of serological IL-34. Then serum IL-34 levels between the SLE group and healthy controls were analyzed by the Mann-Whitney U test. Meanwhile, the correlations between the serum IL-34 levels and disease activity indexes and other established serum markers were assessed. Furthermore, the serum IL-34 levels of 20 active SLE patients were reevaluated when diseases were in the remission stage from corticosteroids or immunosuppressive drugs. Serum IL-34 levels were significantly higher in SLE patients compared to healthy controls. Their levels were remarkably associated with accumulation of the clinical features of SLE. Additionally, IL-34 titers were positively correlated with the SLE disease activity indexes, anti-double-stranded DNA antibody (anti-dsDNA) titers and C-reactive protein (CRP) levels, and inversely with complement3 (C3) levels. Moreover, serum IL-34 levels were significantly decreased after successful treatment of SLE. Serum IL-34 could be a candidate biomarker for SLE as there are elevated serum levels in treatment-naive SLE patients and we saw a significant decrease after effective treatment.

## 1. Introduction

Systemic lupus erythematosus (SLE) is a heterogeneous multi-systemic autoimmune and inflammatory disease characterized by the polyclonal activation of T and B lymphocytes, the production of autoantibodies, and the formation of immune complexes that result in tissue and organ damage and are eventually associated with considerable morbidity and mortality [1,2]. Its diversity of clinical manifestations is matched by the complexity of the factors (genetic, hormonal and environmental) that cause it, and the array of autoantibodies with which it is associated. However, the precise cellular and molecular mechanisms leading to SLE, and real factors that determine which organs are involved, remain poorly understood. Hence, SLE still remains a remarkable and challenging disorder.

Initially identified in 2008, interleukin-34 (IL-34) is the second colony-stimulating factor-1 receptor (CSF-1R) ligand and shares the receptor Fms with macrophage colony-stimulating factor (M-CSF) [3,4]. Notably, IL-34 plays pivotal roles in the proliferation and differentiation of mononuclear phagocyte lineage cells, osteoclastogenesis, development of Langerhans cells and microglia, and inflammation [5,6]. Recent study revealed that IL-34 plays important roles in the pathogenesis of chronic inflammations such as in rheumatoid arthritis (RA) [7,8,9,10]. Both SLE and RA are systemic autoimmune diseases and they share many common features and clinical presentations. Thereby, we made an effort to investigate whether IL-34 is involved in the pathogenesis of SLE.

## 2. Results

### 2.1. The Demographic and Clinical Characteristics of Study Subjects

Taken together, 131 people were enrolled in this study; 78 were SLE patients, and 53 were healthy controls. There were 20 active and 58 inactive patients in the SLE patient group and the duration of the SLE was seven (3.6–12.1) years (median (interquartile range)). From the aspect of age distribution, the ages (median (interquartile range)) of SLE patients and healthy controls were 55.2 (48.1–66.7) years and 57.6 (45.5–66.1) years, respectively. The sex ratios were nine males and 69 females, and six males and 47 females for the SLE patient and healthy groups, respectively. There were no significant differences between SLE patients and controls in terms of gender and age. However, there were significant differences between SLE patients and healthy controls with regard to levels of anti-dsDNA Ab, C3, C4, CRP and ESR (*p* < 0.001) (Table 1).

### 2.2. The Serum IL-34 Levels Were Elevated in Patients with SLE

**Figure 1:**
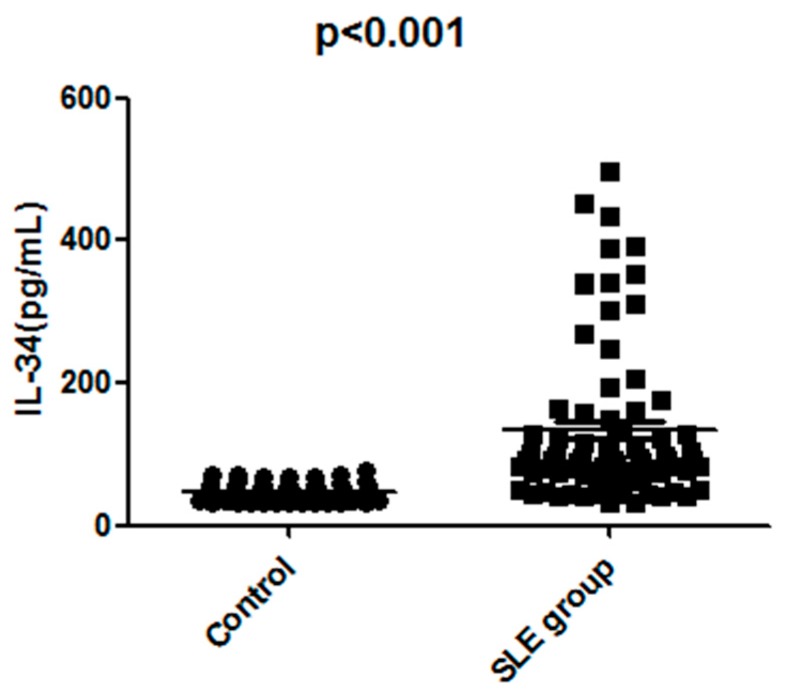
Scatter-plots of serum interleukin-34 (IL-34) levels in healthy control subjects and SLE (systemic lupus erythematosus) patients. The horizontal lines indicate the median concentration for each group. The differences between SLE patients and controls were determined by non-parametric Mann-Whitney rank sum test.

**Figure 2:**
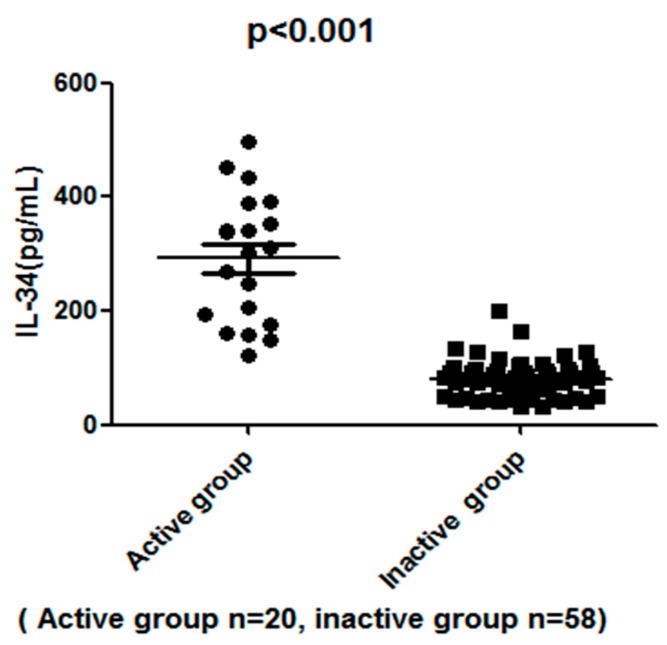
Scatter-plots of serum interleukin-34 (IL-34) levels in active SLE (systemic lupus erythematosus) patients and inactive SLE patients. The horizontal lines indicate the median concentration for each group. The differences between SLE patients and controls were determined by non-parametric Mann-Whitney rank sum test.

Firstly, we compared serum IL-34 levels between 78 SLE patients and 53 healthy controls in order to investigate the role of IL-34 in the pathogenesis of SLE. Serum IL-34 levels of SLE patients (median, 128.9 pg/mL) were significantly higher than those of healthy controls (median, 52.4 pg/mL; *p* < 0.001) (Figure 1). Likewise, serum IL-34 levels of active SLE patients (median, 312 pg/mL) were markedly higher than those of inactive patients (median, 97 pg/mL; *p* < 0.001) (Figure 2). Moreover, the levels of serum IL-34 in the controls kept within the range of 20 to 80 pg/mL.

### 2.3. The Serum IL-34 Levels in SLE Patients Correlated with Disease Activities

**Figure 3:**
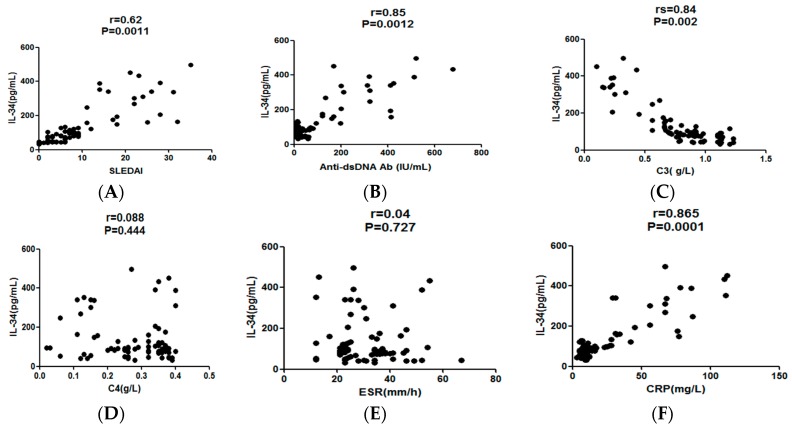
(**A**) Correlation of serum IL-34 concentrations with systemic lupus erythematosus disease activity index (SLEDAI) in all systemic lupus erythematosus (SLE) patients (*n* = 78). Spearman’s rank correlation coefficient was employed to assess correlations; *p*-values are shown; (**B**) Correlation of serum IL-34 concentrations with anti-double-stranded DNA antibody (anti-dsDNA Ab) in all SLE patients (*n* = 78). Spearman’s rank correlation coefficient was employed to assess correlations; *p*-values are shown; (**C**) Correlation of serum IL-34 concentrations with complement 3 (C3) in all SLE patients (*n* = 78). Spearman’s rank correlation coefficient was employed to assess correlations; *p*-values are shown; (**D**) Correlation of serum IL-34 concentrations with complement 4 (C4) in all SLE patients (*n* = 78). Spearman’s rank correlation coefficient was employed to assess correlations; *p*-values are shown; (**E**) Correlation of serum IL-34 concentrations with erythrocyte sedimentation rate (ESR) in all SLE patients (*n* = 78). Spearman’s rank correlation coefficient was employed to assess correlations; *p*-values are shown; (**F**) Correlation of serum IL-34 concentrations with C-reactive protein (CRP) in all SLE patients (*n* = 78). Spearman’s rank correlation coefficient was employed to assess correlations; *p*-values are shown.

**Figure 4:**
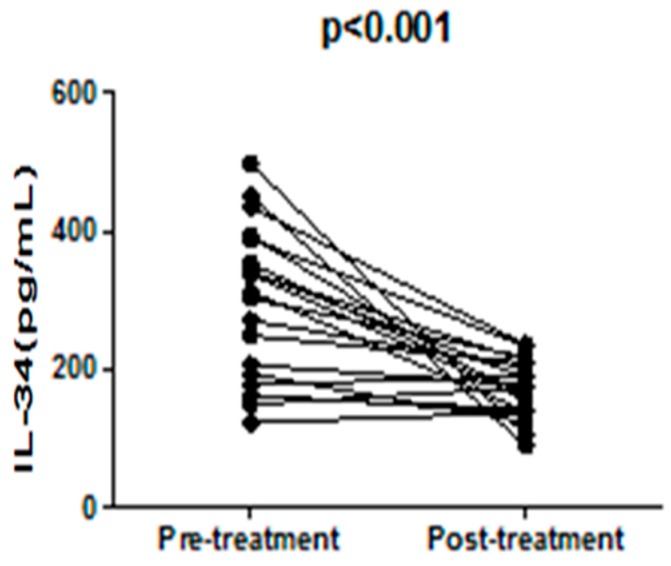
Serum IL-34 levels were significantly decreased after ameliorating the disease with treatment.

Next, we analyzed the relationship between serum IL-34 levels and indicators for SLE disease activity. Consequently, the serum IL-34 levels demonstrated a significantly positive correlation with SLEDAI (rs = 0.62; *p* = 0.0011; Figure 3A). They also positively correlated with the titer of anti-dsDNA Ab (rs = 0.45; *p* = 0.012; Figure 3B) and levels of CRP (rs = 0.65; *p* = 0.013; Figure 3F), but inversely correlated with serum levels of C3 (rs = 0.54; *p* = 0.002; Figure 3C). Moreover, active SLE patients experienced a significant decrease in serum IL-34 levels after disease amelioration by treatment (*p* < 0.001; Figure 4). Thus, serum IL-34 levels reflected disease activities of SLE though there was no significant correlation between serum IL-34 levels and C4 (rs = 0.26; *p* = 0.055; Figure 3D) and ESR (rs = 0.21; *p* = 0.053; Figure 3E).

### 2.4. The High Titers of Serum IL-34 Were Associated with the Accumulation of the Clinical Features of SLE

Finally, we investigated the relation between serum IL-34 levels and SLE-related clinical features. SLE patients were classified into three grades according to the number of clinical features presented. Serum IL-34 levels elevated significantly with the increasing grade of clinical features (*p* < 0.001; Table 2). Therefore, high serum IL-34 levels were associated with the accumulation of the clinical features of SLE.

## 3. Discussion

Recently, interleukin-34 (IL-34) was identified as an alternative ligand of colony-stimulating factor-1 receptor (CSF-1R) which is structurally related to CSF-1 but bears no sequence homology with CSF-1 [11,12]. IL-34 binds to CSF-1R at the cleft between D2 and D3 [13]. Besides, IL-34 could bind especially to the extracellular domain of receptor-type protein-tyrosine phosphataseζ (PTP-ζ) [14] and chondroitin sulphate [15].

IL-34 promotes the differentiation and survival of monocytes and macrophages which are the predominant infiltrating cell types in the inflamed synovium and produce inflammatory cytokines such as tumor necrosis factor (TNF) α and interleukin-6 (IL-6) [16]. Besides, IL-34 itself was shown capable of inducing proinflammatory cytokines and chemokines such as IL-6 and interleukin-8 (IL-8) [17]. Furthermore, accumulating evidence suggested that the CSF-1R pathway played a crucial role in chronic immune disorders such as RA, Sjogren’s syndrome (SS) [18] and lupus nephritis (LN) [19]. However, whether IL-34 is involved in the immunopathogenic process of systemic lupus erythematosus (SLE) is still unexplored in animal models and cell experiments. Therefore, we conducted this clinical study to investigate whether IL-34 is involved in the pathogenesis of SLE. Our study confirmed that serum IL-34 levels are significantly elevated in SLE patients, especially in active SLE patients. This is the first time that our study brings insight into the relationship between serum IL-34 levels and SLE disease activities, as well as clinical features. For example, serum IL-34 levels correlated with SLEDAI, anti-dsDNA Ab, CRP and C3. In particular, SLEDAI was most frequently used to assess SLE disease activity [20]. CRP is the acute-phase reaction protein and is elevated in most active SLE patients. Therefore, the IL-34-SLEDAI and IL-34-CRP correlations suggested that IL-34 is involved in SLE evolution. Likewise, anti-dsDNA antibodies are essential to SLE pathology. Thus, IL-34- anti-dsDNA Ab indicated that IL-34 may play an important role in the pathogenesis of SLE. Moreover, serum IL-34 levels decreased significantly after disease amelioration by treatment. Though the sample size of active SLE patients is rather small, it is still plausible to hypothesize that IL-34 could be a useful biomarker for SLE disease activity and therapeutic effects. These findings also shed new light on the dysregulation of the immune system in autoimmune diseases.

Still, the length of follow-up was not long enough, so this study could not analyze the relationship between IL-34 and the long-term complications and survival parameters of SLE.

In conclusion, this study illustrated that the serum IL-34 levels were elevated in patients with SLE and were associated with lupus disease activity. It is likely that IL-34 is involved in the pathogenesis of SLE and could be a potential biomarker for SLE disease activity.

## 4. Materials and Methods

### 4.1. Subjects

We performed a cross-sectional study. All SLE patients were consecutively recruited at The First Affiliated Hospital of Chongqing Medical University between the years of 2014 and 2015. Meanwhile, the healthy staff members for control were recruited from the physical examination center of The First Affiliated Hospital of Chongqing Medical University. Totally, 78 SLE patients and 53 healthy volunteers were enrolled. All of the SLE patients met at least four of the American College of Rheumatology (ACR) revised criteria [21]. Besides, we excluded SLE patients and healthy control with other autoimmune and infectious diseases, tumor, diabetes and obesity [2]. The SLE disease activity was measured according to the SLE Disease Activity Index (SLEDAI) and patients with SLEDAI score ≥6 were defined as active SLE [22].

Each SLE patients received a standardized medical history collection and physical examination. The serum samples of active and inactive SLE patients were collected before the initiation or reinforcement of treatment and during regular hospital visits respectively. The active SLE patients were treated with corticosteroids or immunosuppressive drugs initial evaluations. Specifically, the serum IL-34 levels of active SLE patients were reevaluated after effective treatment. Meanwhile, control sera were obtained from healthy staff members (*n* = 53). All the serum samples were preserved at −20 °C.

This study was approved by the ethics committee of our institution, and informed consent was obtained from all participants.

### 4.2. Data Collection

The information of SLE patients covered demographic data, such as age, sex, disease duration, clinical manifestations, and laboratory values. SLE-related features included malar rash/discoid rash, oral or nasal ulcers, alopecia, serositis, arthritis, active nephritis, CNS (central nervous system) lupus, vasculitis, fever >38 °C, thrombocytopenia, leukopenia, and anemia and were based on the SLEDAI except anemia [12]. For instance, anemia, thrombocytopenia and leukopenia were defined as a decrease in the concentration of hemoglobin to <100 g/L, in the number of platelets to <100 × 10^9^/L, and in the number of white blood cells to <3 × 10^9^/L. Moreover, the laboratory values of serum anti-double stranded DNA (anti-dsDNA) antibodies of SLE patients were measured.

### 4.3. Methods of Measurements

Turbidimetric immunoassay on Beckman Immage 800 immunology analyzer was employed to measure the serum C3 and C4 and C-reactive protein (CRP) levels. The levels of serum anti-dsDNA antibodies were measured by using fluorescence-enzyme immunoassay (Euroummun, Lubeck, Germany). The erythrocyte sedimentation (ESR) levels were manually performed via Westergren method. The serum IL-34 levels were determined by using enzyme linked immunosorbent assay (ELISA) kits (R&D Systems, Minneapolis, MI, USA). The operation processes were performed in strict accordance with the manufacturer’s protocol.

### 4.4. Statistical Analysis

All statistical analysis were achieved by SPSS 17.0. The index data of SLE patients and control group is skewed distribution (the Kolmogorov-Smirnov Test), so that Mann-Whitney U test was employed to analyzed the differences between two groups. The relations between IL-34 levels and other continuous variables were analyzed by using the Mann-Whitney U test as well. The IL-34 levels before and after treatment were compared by using a paired t test. Furthermore, the SLE patients were divided into three groups based on scores of clinical features (0, 1 to 2, and 3 to 8). Among three groups, *p*-values were calculated by analysis of variance and were adjusted by use of the Bonferroni correction. *p*-Values < 0.05 were considered significant.

## Figures and Tables

**Table 1:** Demographic and clinical characteristics of the study subjects.

Features	Healthy Controls (*n* = 53) Mean (95% Confidence Interval)	SLE Group (*n* = 78)
Age, years (edian (IQ range))	57.6 (45.5–66.1)	55.2 (48.1–66.7)
Male/female	6/47	9/69
Disease duration, years (median (IQ range))	No	7 (3.6–12.1)
Anti-dsDNA Ab, IU/mL (median (IQ range))	14 (9–22)	30.1 (7.9–678) *
IL-34, pg/mL (median (IQ range))	41.2 (31.3–77)	91.3 (32.1–498.4) *
ESR, mm/h (median (IQ range))	7 (5–15)	26 (12.0–67.0) *
CRP, mg/L (median (IQ range))	<9	11.0 (3.0–112.0) *
C3, g/L (median (IQ range))	1.21 (0.79–1.32)	0.8 (0.1–1.23) *
C4, g/L (median (IQ range))	0.25 (0.21–0.38)	0.32 (0.02–0.4) *

Anti-dsDNA Ab, anti-double-stranded DNA antibody; IL-34, interleukin-34; ESR, erythrocyte sedimentation rate; CRP, C-reactive protein; C3, complement 3; C4, complement 4. IQ, interquartile. * *p* < 0.001 vs. control

**Table 2:** Associations between SLE clinical features and titer of IL-34.

Clinical Features	Number	Median IL-34 (95% Confidence Interval)
0	14	43 (30.1–50.3) *
1–2	44	124.2 (50.5–202.4) *
3–8	20	312.0 (193.5–498.4) *

Number of positive clinical features including malar rash/discoid rash, alopecia, oral or nasal ulcers, serositis, arthritis, active nephritis, CNS (central nervous system) lupus, vasculitis, temperature > 38 °C, thrombocytopenia, leukopenia, anemia. * *p* < 0.001, Bonferroni test was employed to compared the three clinical feature groups.
